# Clinical Factors Influencing Phenotype of HCMV-Specific CD8+ T Cells and HCMV-Induced Interferon-Gamma Production after Allogeneic Stem Cells Transplantation

**DOI:** 10.1155/2013/347213

**Published:** 2013-02-02

**Authors:** Inmaculada Gayoso, Sara Cantisán, Carolina Cerrato, Joaquín Sánchez-García, Carmen Martin, Rafael Solana, Antonio Torres-Gomez, Julian Torre-Cisneros

**Affiliations:** ^1^Instituto Maimonides para la Investigacion Biomedica de Córdoba (IMIBIC), Reina Sofia University Hospital, 14004 Cordoba, Spain; ^2^Department of Infectious Diseases and IMIBIC, Reina Sofia University Hospital, Avenida Menéndez Pidal s/n, 14004 Cordoba, Spain; ^3^Spanish Network for Research in Infectious Diseases (REIPI), IMIBIC, Reina Sofia University Hospital, Avenida Menéndez Pidal s/n, 14004 Cordoba, Spain; ^4^Department of Haematology and IMIBIC, Reina Sofia University Hospital, Avenida Menéndez Pidal s/n, 14004 Cordoba, Spain; ^5^Department of Immunology and IMIBIC, Reina Sofia University Hospital, Avenida Menéndez Pidal s/n, 14004 Cordoba, Spain

## Abstract

Human cytomegalovirus (HCMV) infection causes significant morbidity and mortality after hematopoietic stem cell transplantation (HSCT). In this work, we characterized the phenotype and interferon-gamma (INF-*γ*) production of HCMV-specific T cells using QuantiFERON-HCMV assay in 26 patients 6 months after HSCT. We analysed whether these two parameters were associated with clinical variables. Our results showed that the patients receiving stem cells from donors ≥40 years old were 12 times more likely to have HCMV-specific CD8+ T cells with “differentiated phenotype” (CD45RA+CCR7+ ≤6.7% and CD28+ ≤30%) than patients grafted from donors <40 years old (OR = 12; *P* = 0.014). In addition, a detectable IFN-*γ* production in response to HCMV peptides (cutoff 0.2 IU/mL IFN-*γ*; “reactive” QuantiFERON-HCMV test) was statistically associated with HCMV replication after transplantation (OR = 11; *P* = 0.026), recipients ≥40 versus <40 years old (OR = 11; *P* = 0.026), and the use of peripheral blood versus bone marrow as stem cell source (OR = 17.5; *P* = 0.024). In conclusion, donor age is the only factor significantly associated with the presence of the “differentiated phenotype” in HCMV-specific CD8+ T cells, whereas HCMV replication after transplantation, recipient age, and stem cell source are the factors associated with the production of IFN-*γ* in response to HCMV epitopes.

## 1. Introduction

Human cytomegalovirus (HCMV) infection is a major cause of morbidity and mortality in subjects who undergo allogeneic stem cell transplantation (HSCT) due to the long period of immunodeficiency after SCT [1–3]. HCMV-specific immune reconstitution after HSCT plays a critical role in preventing HCMV infection and disease. Lack of this T-cell HCMV-specific subpopulation is associated with a higher risk of HCMV infection, as has been reported in HCMV-seropositive patients receiving an HSCT from HCMV-seronegative donors [4–8]. The magnitude of HCMV-specific CD8+ T-cell recovery predicts the risk of progressive HCMV infection [8, 9], but HCMV replication after HSCT also depends on the presence of dysfunctional HCMV-specific CD8+ T cells rather than on the absolute numbers of HCMV-specific T cells [10, 11].

After encountering HCMV, naive T cells proliferate and become effector memory HCMV-specific CD8+ T cells, which exert an effector function in peripheral tissues and exhibit a differentiated phenotype. During this process, the downregulation of some costimulatory surface molecules (such as CD28 or CD27) and an increase in interferon-gamma (IFN-*γ*) production have been reported [12–15]. Therefore, a decrease in the percentage of naive T cells and a parallel increase in the percentage of differentiated effector memory T cells (mostly CD28−) are observed after HCMV infection. In addition, these phenotypic changes on HCMV-specific CD8+ T cells have also been linked with immunosenescence, suggesting that HCMV accelerates the process of age-associated immune function impairment [16–19]. In the HSCT setting, a correlation between the absolute number of circulating CD8+ HCMV-specific T cells in the recipient and the risk of HCMV infection has been demonstrated [8]. In addition, the number of less-differentiated CD8+ HCMV-specific T cells in the donor is associated with protection of HCMV infection. These donor lymphocytes acquire a differentiated profile and restricted function in the recipient after HSCT [10, 11]. Therefore, assessment of the maturation status and functional capability of circulating HCMV-specific T lymphocytes after HSCT and the impact of clinical factors in the HCMV-specific immune recovery is crucial to improving our management of HCMV infection in these patients.

In this work, we have studied IFN-*γ* production in response to HCMV peptides and the phenotype of HCMV-specific CD8+ T cells in a group of HSCT patients 6 months after allogeneic transplantation. In this cross-sectional study, we analyse whether these two parameters are associated with HCMV replication after transplantation as well as other clinical variables such as donor and recipient age, donor and recipient serostatus, and stem cell source. Our results show that the differentiated phenotype in HCMV-specific CD8+ T cells was associated only with increased donor age whereas IFN-*γ* production in response to HCMV peptides was associated with HCMV replication, and also with recipient age and stem cell source.

## 2. Materials and Methods

### 2.1. Study Population

Twenty-six HLA-A*0201 patients who received allogeneic HSCT were recruited and peripheral blood samples were drawn at a median of 950 days after HSCT (range 240–2436). Patients underwent HSCT at the Department of Haematology of the Reina Sofia University Hospital (Cordoba, Spain).

### 2.2. HCMV Monitoring and Preemptive Therapy

Plasmatic HCMV viral loads were routinely screened using a Cobas Amplicor HCMV Monitor (Roche Diagnostics, Basel, Switzerland), a commercially available quantitative polymerase chain reaction (PCR) test with a detection limit of 600 copies of HCMVDNA/mL. The prospective monitorization protocol included two determinations per week during the first month or until discharge, and one determination per week until day +100 or +180 in patients with GVHD requiring high-dose steroids. HCMV replication was defined as the presence of any HCMV viral load in plasma over the limit of detection (>600 copies/mL).

Preemptive valganciclovir (Roche, Basel, Switzerland) was administered: (i) at the time of the first positive HCMV viral load in high-risk patients (unrelated donor transplant, steroid treatment) or in patients with a HCMV load ≥ 10.000 copies/mL in a single sample; (ii) at the time of a second positive sample obtained one week after the first. Valganciclovir was administered orally in a dosage of 900 mg b.i.d. for 2 weeks (induction dose) followed by 900 mg qd until negativization of HCMV replication during 2 consecutive weeks (maintenance dose). The dosage was adjusted for creatinine clearance following standard recommendations. Valganciclovir was discontinued temporarily or substituted with foscarnet if necessary in patients with a neutrophil count < 0.5 × 109/L despite the administration of G-CSF.

### 2.3. Transplantation Protocol

The conditioning regimen was myeloablative or reduced intensity conditioning protocol (RIC) in patients aged >50 years or with comorbidities. The myeloablative conditioning regimen consisted of hyperfractionated total body irradiation (13.2 Gy in 8 fractions) plus Cyclophosphamide (60 mg/kg/day for 2 consecutive days), Busulphan (0.8 mg/kg i.v. × 16 doses) plus Cyclophosphamide (60 mg/kg/day for 2 days) or ATG (rabbit, 2.5 mg/kg/day × 4 days) plus Cyclophosphamide (50 mg/kg/day × 4 days). The reduced intensity protocols consisted of Fludarabine (30 mg/m^2^ × 5 days) plus Busulphan (0.8 mg/kg i.v. × 10 doses) or plus Melphalan (70 mg/m^2^ × 2 doses). Acute GVHD prophylaxis varied according to donor type and conditioning regimen intensity: recipients of a matched related transplant after myeloablative conditioning received cyclosporin plus a short course of methotrexate while recipients of reduced intensity conditioning received cyclosporine plus mycophenolate mofetil. In addition, recipients of an unrelated donor graft in remission at the time of HSCT received rabbit ATG (Thymoglobulin, Genzyme, Framingham, MA, USA) 6 mg/kg days −4 to −2.

### 2.4. Phenotype of HCMV-Specific CD8+ T-Cell Population

Blood samples were collected in ACD tubes and peripheral blood mononuclear cells (PBMCs) were obtained by Ficoll-Hypaque-1077 gradient (Sigma, St. Louis, MO, USA). To study the HCMV-specific CD8+ T-cell population, an HLA-A*0201 pentamer bound to an immunodominant HCMV epitope (pp65, NLVPMVATV) APC-labeled (Proimmune, Oxford, UK) was used. PBMCs (10^6^ per tube) were stained with monoclonal antibodies (mAbs) conjugated with the appropriate fluorochrome for 30 min at 4°C according to standard procedures. Cells were also incubated with mAbs anti-CD8 (PerCP), anti-CD45RA (FITC), anti-CCR7 (PE), anti-CD28 (PE), or anti-CD27 (PE) (BD Biosciences, San Jose, CA, USA). The frequency of A2/HCMVpp65 pentamer-positive cells referred to the CD8+ T-cell population. The expression of the different markers referred to A2/HCMVpp65 pentamer-positive cells. For each sample, 10^5^ events were acquired. Sample acquisition and data analysis were performed using a FACSCalibur cytometer (Becton-Dickinson) and FlowJo software (Tree Star Inc., v 7.2.1, Ashland, OR, USA).

### 2.5. INF-*γ*  Production Using the QuantiFERON-HCMV Assay

The QuantiFERON-HCMV test was performed according to the manufacturer's instructions (Cellestis Ltd., Melbourne, Australia). In brief, the assay consists of two steps: an overnight blood culture with HCMV CD8+ T-cell synthetic peptide epitopes and the subsequent quantification of INF-*γ* production by an enzyme-linked immunosorbent assay (ELISA). Initially, 1 mL aliquots of heparinized whole blood were collected in three QuantiFERON-HCMV blood collection tubes that were shaken vigorously for 5 sec. The tubes contained either (i) a mix of 21 human cytomegalovirus (HCMV) peptide epitopes from a variety of HCMV proteins including pp65, IE-1, IE-2, pp50, and gB (1 *μ*g/mL of 21 different peptides), (ii) no antigens, only sterile phosphate buffered saline (negative control), or (iii) phytohemagglutinin (PHA; positive mitogen control). The tubes were incubated for 16–24 h at 37°C. Following incubation, supernatants were harvested and IFN-g levels measured (IU/mL) using a standard ELISA as per manufacturer's instructions. The results were calculated using Analysis Software v 1.51 (Cellestis Ltd.). The cutoff value for HCMV reactivity was 0.2 IU/mL.

### 2.6. Statistical Analysis

Statistical analysis was performed using SPSS software for Windows v.18.0 and EPIDAT 3.1 software. The population of HCMV-specific CD8+ T cells was expressed as a proportion of the total population of CD8+ T cells. The *χ* ^2^ test or Fisher exact test was used to assess the association between independent and dependent variables. A *P* value < 0.05 was considered statistically significant.

## 3. Results

### 3.1. Patient Characteristics

Twenty-six HLA-A*0201 patients who received allogeneic HSCT for hematological malignancies at the Reina Sofia University Hospital were studied. Samples were obtained after lymphocyte recovery (1.000/10^9^/L), at least six months following HSCT (median 950 days; range 240–2436 days). The characteristics of the subjects in the study are summarized in Table 1. Median recipient age was 42 years old (range 16–68) and median donor age was 40 years (range 11–64). Fifty percent of the patients received a “nonmyeloablative” conditioning regimen. Fourteen patients received peripheral blood stem cells (53.8%) and 12 from bone marrow stem cells (46%). Fourteen HSCTs were donor (D) and recipient (R) HCMV-seropositive (D+/R+), 10 were D−/R+ and 2 were D−/R−. Sixty-nine percent of patients received corticosteroids after HSCT. Eleven patients (42.3%) experienced graft-versus-host disease (GVHD) and 15 (57.7%) had HCMV replications after HSCT but none of them underwent HCMV disease. Sixty-nine percent of donors were HLA-matched related, 23% matched unrelated, and only 7% mismatched.

### 3.2. Classification of Patients According to the Phenotype of HCMV-Specific CD8+ T Cells

We used HLA-A*0201(pp65) pentamers to study the frequency of the HCMV-specific subpopulation and the expression of CCR7, CD45RA, and CD28 on this subset in HSCT patients. Our results showed that the percentage of CD8+ T cells was 35.1% ±14.8 and the percentage HCMV-specific over total CD8+ T cells was 1.36% ± 2.1. The phenotypic analysis showed that the mean percentage of HCMV-specific CD8+ T cells with naive phenotype (CD45RA+CCR7+) was 6.7% ± 11.3 and the mean percentage of CD28 expression on these cells was 30% ± 23.2.

**Figure 1:**
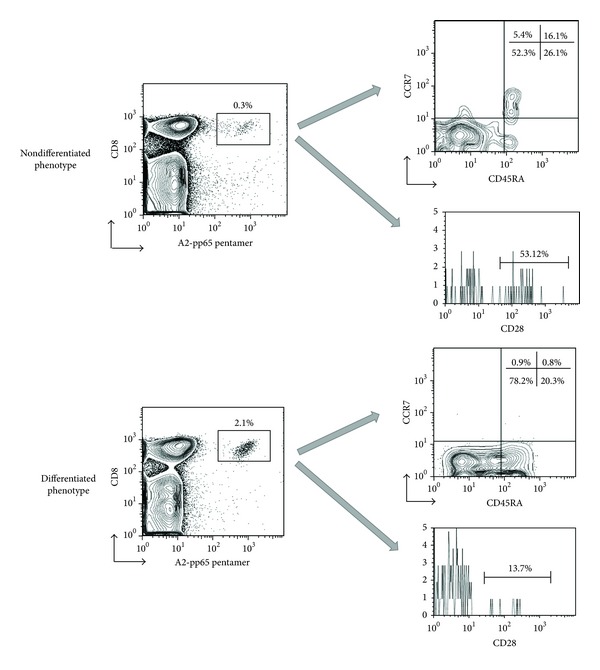
Quantitative assessment of HCMV-specific T cell with “differentiated phenotype.” Quantitative assessment of HCMV-specific CD8+ T cells using A2-pp65 pentamer and monoclonal antibodies specific for CD8. The phenotype of HCMV-specific CD8+ T cells was determined analyzing the expression of CCR7, CD45RA, and CD28. A representative example is shown.

Considering these values, patients were classified into two groups: those having a “differentiated phenotype” if the HCMV-specific CD8+ T cells had values of CD45RA+CCR7+ naive cells ≤6.7% and CD28+ cells ≤30% and those having a “nondifferentiated phenotype” if they did not fulfill these criteria. A representative example is shown in Figure 1.

### 3.3. The Differentiated Phenotype of HCMV-Specific CD8+ T Cells in the Recipient Is Associated with Donor Age

As shown in Table 2, donor age was the only factor significantly associated with the presence of the “differentiated phenotype” in HCMV-specific CD8+ T cells in the recipient (OR = 12; *P* = 0.014), whereas recipient's age, stem cell source or other HCMV-related factors were not statistically significant. Thus, HSCT patients receiving stem cells from donors older than 40 years are 12 times more likely to have HCMV-specific CD8+ T cells with differentiated phenotype than patients grafted from donors younger than 40 years old.

### 3.4. INF-*γ*  Production in Response to HCMV Peptides Is Associated with Post-HSCT HCMV Replication, Recipients' Age, and Stem Cell Source

The patients were divided into two groups according to INF-*γ* production in response to specific HCMV peptides using QuantiFERON-HCMV assay: “reactive” patients in whom the INF-*γ* production was higher than 0.2 IU/mL and “nonreactive” patients in whom the INF-*γ* production was lower. The results shown in Table 3 demonstrate a statistically significant association with HCMV replication after transplantation (OR = 11; *P* = 0.026), recipients' age (OR = 11; *P* = 0.026), and the stem cell source (OR = 17.5; *P* = 0.024). Hence, recipients who have experienced HCMV replication after HSCT, those older than 40 and those grafted with peripheral blood are more likely to harbour circulating effector HCMV-specific CD8+ T cells producing INF-*γ*. We did not find any other significant difference although the association with donor HCMV serology was close to statistical significance (*P* = 0.074). Although a multivariate analysis would be of help to better understand these associations, the relatively small sample size precludes doing it.

## 4. Discussion

In this work, we have studied clinical factors that have impact on T-cell phenotype and responses to HCMV in a cross-sectional study of 26 HLA-A*0201 HSCT recipients. Our results show that the “differentiated phenotype” (levels of HCMV-specific CD8+ T cells CCR7+CD45RA+ ≤6.7% and CD28+ ≤30%) is statistically associated only with donor age older than 40 years. A previous study [20] has been shown that HCMV-specific T cells after HSCT mostly correspond to the expansion of HCMV-specific T cells from donors. The fate of these cells, which are adoptively transferred during HSCT, is determined by their characteristics in the donor rather than by other factors related to transplantation or virus reactivation. Furthermore, the authors showed that HCMV-specific T cells with a differentiated phenotype (CD27−CD57+) in the donor tended to increase in the recipient whereas less differentiated memory cells (CD27+CD45RO+CD57−) tended to decrease. In the study, the authors did not analyse factors associated with the percentage of naive or memory cells in either the donor or the recipient. However, we and others have shown that age is one of the major factors that determine the percentage of HCMV-specific CD8+ T cells and their phenotype [21] in healthy individuals. Thus, the association between the “differentiated phenotype” and donors' age observed in our series likely reflects the phenotype of the adoptively transferred donor cells. This observation is of interest since it has been shown that HCMV-specific T-cell immunity after HSCT is largely determined by the frequency and phenotype of HCMV-specific T cells from the donors [20, 22], which are affected by ageing [17, 21, 23–25].

In addition, we have studied IFN-*γ* production in response to HCMV peptides in these patients. Our results show that IFN-*γ* production (“reactive” QuantiFERON-HCMV assay) is associated with HCMV replication, recipients' age, and stem cell source.

HCMV reactivation after HSCT is lower in patients with higher levels of HCMV-specific T cells with a terminally differentiated phenotype (CD45RO−CD62L−) in the donor graft [22]. Protection from HCMV reactivation has been associated to proliferation of HCMV-specific T cells [1] and especially to high levels of HCMV-specific CD8+ T cells secreting INF-*γ* [26]. In kidney transplants receptors, Egli et al. [27] have shown differences in IFN-*γ* responses to HCMV-pp72 versus HCMV-pp65. The latter appeared to be better suited to interrogate the CD4 as well as the CD8 T-cell compartment in a ROC analysis, and associated a higher pp65-specific CD4 T-cell response with protection from CMV replication. Furthermore, protection against HCMV replication is associated to the presence of HCMV-specific T cells with that have the capacity to produce several cytokines in response to HCMV peptide (dual function IL2/IFN-*γ* or polyfunctional) whereas the presence of T cells that produced only IFN-*γ* was considered a sign of functionally exhausted T cells unable to efficiently control HCMV replication [28].

Our results showing the association of HCMV replication with IFN-*γ* production in response to HCMV peptides using the QuantiFERON-HCMV assay could suggest that IFN-*γ* production is insufficient to control HCMV replication after HSCT as suggested by Krol et al. [28], although it cannot be discarded that IFN-*γ* production is the consequence of the expansion and differentiation of IFN-*γ* producing HCMV-specific T cells in response to HCMV replication.

We also show that recipient's age is associated with IFN-*γ* production in response to peptides. Patient's age has a profound significance in HCMV replication as patients older than 40 have a higher probability of HCMV replication than younger patients, even if they had similar percentages of HCMV-specific T cells secreting IFN-*γ* [26].

Several studies have analysed differences in immune reconstitution by comparing different stem cell sources. Bone marrow and peripheral blood contain different ratios of naive and effector-memory T-cell subsets [29–31], which could explain the lower number of infections in recipients grafted with peripheral blood stem cells [32]. HSCT using peripheral blood stem cells exhibited better recovery of HCMV-specific CD8+ T-cell function 3 months after transplantation compared to patients that received bone marrow stem cells [10], thus supporting that differences in INF-*γ* production by distinct T-cell subpopulations lead to different HCMV infection rates. In contrast, a recent study [33] reported a higher incidence of HCMV infection and disease one month after peripheral blood stem cell transplantation that correlated with the lower functional capacity of HCMV-specific T cells, in particular with an increased proportion of HCMV-specific CD8 T cells that produced a single cytokine and a lower proportion of polyfunctional cells. After 3 months, however, HCMV-specific responses were similar between recipients of bone marrow or peripheral blood as the stem cell source. Our results showing that the use of peripheral blood associates with increased levels of IFN-*γ* production in response to HCMV peptides strongly suggest that peripheral blood contains higher proportions of HCMV-specific T cells with a capacity to release IFN-*γ* in response to HCMV than bone marrow. However, as indicated above, IFN-*γ* production is insufficient to control HCMV replication. This incapacity to control HCMV could be due to the presence in these patients of HCMV-specific functionally exhausted T cells that produce only IFN-*γ* in response to HCMV as suggested by Krol et al. [28].

## 5. Conclusions

In summary, our results indicate that whereas the phenotype of HCMV-specific CD8+ T cells after HSCT is associated with donor age, IFN-*γ* production in response to HCMV peptides associates with HCMV replication episodes, recipient age, and the use of peripheral blood as the stem cell source. The association of IFN-*γ* production in response to HCMV peptides with HCMV replication episodes and peripheral blood as the source of stem cells supports that posttransplant HCMV replication induces the expansion of IFN-*γ* producing HCMV-specific T cells present in the donor's peripheral blood, contributing to the control of HCMV infection. However, it cannot be excluded that *in vitro* IFN-*γ* production is insufficient to control HCMV replication after HSCT and that it might reflect the presence of “monofunctionally” exhausted rather than “polyfunctional” effector-memory specific T cells [28]. It could be of interest to develop other tests easy to perform, economical and reliable to immunomonitor polyfunctional HCMV-specific T cells, which will likely correspond better to HCMV protection. In addition, since the observations reported here are derived from a cross-sectional study with a relatively small sample size, these findings cannot be generalized based on this study alone. Thus, it will be important to confirm and extend these findings in a prospective study that include the analysis of HCMV-specific T cells before HSCT and other additional time points. Such a study would help clinicians to assess the risk of HCMV infection as well as immunotherapy strategies for the reconstitution of HCMV-specific T-cell immunity and the subsequent control of HCMV infection after HSCT.

## Figures and Tables

**Table 1:** Clinical characteristics of the study population.

Characteristics	Data
Patients (number)	26
Recipient age (years): mean (SD)	40 (13.5)
Donor age (years): mean (SD)	40 (15)
Recipient sex, male/female, (*n*, (%))	15 (57.7)/11 (42.3)
Donor sex, male/female (*n*, (%))	20 (76.9)/6 (23.1)
Disease (*n*, (%))	
AML	5 (19.2)
ALL	2 (7.6)
CML	3 (11.5)
Non-Hodgkin's lymphoma	4 (15.3)
MM	4 (15.3)
Aplasia	2 (7.6)
MDS	3 (11.5)
PNH	3 (11.5)
Conditioning regimen (*n*, (%))	
NMA	13 (50)
MA	13 (50)
Stem cell source (*n*, (%))	
Bone marrow	12 (46.1)
Peripheral blood	14 (53.8)
Pretransplant HCMV donor serostatus/recipient (*n*, (%))	
D+/R+	14 (53.8)
D−/R+	10 (38.4)
D−/R−	2 (7.7)
HCMV replication after transplant (*n*, (%))	
Yes	15 (57.7)
No	11 (42.3)
HLA matching (*n*, (%))	
Matched related	18 (69.2)
Matched unrelated	6 (23.1)
Mismatched unrelated	2 (7.7)
GVHD (*n*, (%))	
Yes	11 (42.3)
No	15 (57.7)
Corticosteroids (*n*, (%))	
Yes	18 (69.2)
No	8 (30.8)
Frequency of CD8+ T cells (mean, SD)	35.1 (14.8)
Frequency of HCMV-specific CD8+ T cells (mean, SD)	1.4 (2.1)
Frequency of HCMV-specific CD8+ T cells naive (mean, SD)	6.7 (11.3)
Frequency of HCMV-specific CD8+ T cells CD28+ (mean, SD)	30 (23.2)

AML: acute myeloid leukemia; ALL: acute lymphatic leukemia; CML: chronic myeloid leukemia; MM: multiple myeloma; MDS: myelodysplastic syndrome; PNH: paroxysmal nocturnal hemoglobinuria; NMA: non-myeloablative; MA: myeloablative. D: donor; R: recipient; HCMV: human cytomegalovirus; GVHD: graft-versus-host disease. Naive cells are CD45RA+CCR7+.

**Table 2:** Univariate analysis of factors associated with “differentiated phenotype” in CD8+ HCMV-specific T cells.

Parameter	All patients	Differentiated phenotype (*n* = 16)	Nondifferentiated phenotype (*n* = 10)	Univariate	
				OR (95% CI)	*P* value
Posttransplant HCMV replication (*n*, (%))					
Yes	15 (57.7)	11 (73.3)	4 (26.6)	3.3 (0.6–17.2)	0.228
No	11 (42.3)	5 (45.4)	6 (54.5)		
Donor age (*n*, (%))					
≥40	14 (53.8)	12 (85.7)	2 (14.3)	12 (1.4–85.2)	0.014
<40	12 (46.2)	4 (33.3)	8 (66.7)		
Recipient age (*n*, (%))					
≥40	15 (57.7)	11 (73.3)	4 (26.6)	3.3 (0.6–17.2)	0.228
<40	11 (42.3)	5 (45.4)	6 (54.5)		
Donor HCMV serology (*n*, (%))					
Positive	14 (53.8)	11 (78.6)	3 (21.4)	5.1 (0.9–28.6)	0.105
Negative	12 (46.2)	5 (41.6)	7 (58.3)		
Recipient HCMV serology (*n*, (%))					
Positive	23 (88.5)	16 (69.6)	7 (30.4)	15.4 (0.7–337.2)	0.110
Negative	3 (11.5)	0 (0)^a^	3 (100)		
Stem cell source (*n*, (%))					
Peripheral blood	14 (53.8)	10 (71.4)	4 (28.6)	2.5 (0.5–12.6)	0.420
Bone marrow	12 (46.2)	6 (50)	6 (50)		

OR: odds ratio. CI: confidence interval. “Differentiated phenotype” refers to patients with CD8+ T HCMV-specific cells, naive phenotype (CD45RA+CCR7+) ≤ 6.7% and CD28+ ≤ 30%. These cutoff values are the mean of all patients. *P* values < 0.05 were considered statistically significant.

^a^We have added 0.5 to categories with 0 individuals using EPIDAT 3.1 software.

**Table 3:** Univariate analysis of factors associated with “INF-*γ*” production.

Parameter	All patients	QuantiFERON-HCMV assay		Univariate	
		Reactive (*n* = 14)	Nonreactive (*n* = 8)	OR (95% CI)	*P* value
Post-SCT HCMV replication (*n*, (%))					
Yes	13 (59.1)	11 (84.6)	2 (15.4)	11 (1.4–85.2)	0.026
No	9 (40.9)	3 (33.3)	6 (66.7)		
Donor age (*n*, (%))					
≥40	12 (54.5)	9 (75)	3 (25)	3 (0.5–16.2)	0.378
<40	10 (45.5)	5 (50)	5 (50)		
Recipient age (*n*, (%))					
≥40	13 (59.1)	11 (84.6)	2 (15.4)	11 (1.4–85.2)	0.026
<40	9 (40.9)	3 (33.3)	6 (66.7)		
Donor HCMV serology (*n*, (%))					
Positive	12 (54.5)	10 (83.3)	2 (16.7)	7.5 (1–54.1)	0.074
Negative	10 (45.5)	4 (40)	6 (60)		
Recipient HCMV serology (*n*, (%))					
Positive	19 (86.4)	14 (73.7)	5 (26.3)	16.8 (0.8–418.6)	0.089
Negative	3 (13.6)	0 (0)^a^	3 (100)		
Stem cell source (*n*, (%))					
Peripheral blood	11 (50)	10 (90.9)	1 (9.1)	17.5 (1.6–191.9)	0.024
Bone marrow	11 (50)	4 (36.4)	7 (63.6)		

OR: odds ratio. CI: confidence interval. “Reactive” patients have an INF-*γ* production higher than 0.2 IU/mL. “Nonreactive” patients have less than 0.2 IU/mL. *P* values < 0.05 were considered statistically significant.

^a^We have added 0.5 to categories with 0 individuals using EPIDAT 3.1 software.
